# Quackery Rampant

**Published:** 1896-08

**Authors:** 


### Quackery Rampant.

Dr. R. Menger, of San Antonio, sends the "Red Back" the following, printed on a dodger 4x8, embelished with the picture of the "phenomenon," "a slender, black-haired biped, with a brand new stovepipe hat on the summit of his cranium," Dr. Menger says, and requests us to publish it. As we are now giving some attention to the various forms of quackery which flourish perennially in this genial clime, where there are no legal barriers worth mentioning, we comply with Dr. Menger's request, giving the advertisement as a specimen of a new and hitherto unknown variety, named by himself (or itself), the "phenomenon." We do so to give further emphasis to the urgent need of proper medical legislation. That quackery can not be entirely prevented so long as colleges will grant diplomas to men who will disregard every obligation, and at once engage in the practices of the charletan under the cloak of the college name and degree, is admitted; but there is no doubt, if the medical profession will ask for a bill to prevent the indiscriminate practice of medicine, stating that by practice is not meant the homeopathic practice, the request will be granted, and thus much evil can be prevented. Now, this fellow, if he has a diploma, can not be reached by any law; no law can make a man honest, no law can make a man a gentleman, and no law can make a reputable physician out of a born quack. The great trouble in Texas is that too many of the quacks are graduates, have diplomas; they are, as our S. W. Houston contemporary says, "inside the pasture," and can not be turned out.

Here is the way this one does it (printed in heavy display caps):

"The wonderful magnetic and electric healer, Dr. P. C. K———. Reads disease by sight. Looks into your body and finds your complaints. The new process is equal to the X-rays discovery. Cures guaranteed to the afflicted. The people say this doctor is simply wonderful, and to miss a consultation with him is a great injustice to your health and welfare through life. You who are suffering go and see him at once. He cures all diseases without the aid of medicine. He can tell your complaint by looking you in your face and laying his hands upon yours. Come and consult this wonderful phenomenon and be convinced. Office: No. 517½ L. Commerce st. Office hours from 8 a. m. to 7 p. m. Near Patrick's drug store, Halamuda block."

Dr. Menger says:

"For the fun of it you may publish the enclosed letter, *verbatim*, with the address, as also these lines. I had reported this imposter last year to the authorities on account of the fellow refusing to sign his name to a death certificate of a patient he had been hoodooing for consumption. Our laws on the matter being inadequate he was not punished, also 'because he did not prescribe any medicine,' and I heard no more of him until receiving the above 'ad.' recently. I will try to scare the wits out of him again soon. Just think of the impudence of this 'phenomenon' to address this letter to me after he had been given a lesson on legal practice of medicine before."

In connection with the subject of quacks and quackery, the following letter from an eminent physician, a subscriber, living in North Texas, will be read with interest:

"*Editor Texas Medical Journal:*

"One of our attorneys here answered a personal in the *Gazette* "How to become a lawful physician." I beg to inclose you herewith what the above attorney received in reply. You have probably seen more literature from the same source.

"Knowing your inveterate hatred of all fraud and quackery, and the relentless war you have always waged against them, I take the liberty of mailing this trash to you.

"Their scheme is a good one, trading on the ignorance and prejudice of the people.

"Show me a physician who has been in practice six months and not met the 'old woman,' 'aunt somebody,' and I will show you a doctor who has never had a serious case.

"I think if the 'Red Back' will fire a shot or two at these people they will not do much further harm in Texas."

Here is what they are doing it on:

### IMPORTANT TO UNDERGRADUATES.

Many otherwise splendidly equipped physicians are heavily handicapped by the fact that they have no diploma, and have never received the degree of M. D. This fact leaks out, often the report is started by some competitor who may have no qualification but a sheep-skin, and the doctor who has no diploma finds his business seriously crippled. Nine times out of ten the cause of this is the rumors we have mentioned. Had the doctor been wise enough to have a neat diploma hanging where his customers could get an occasional glimpse of it, these rumors would all have been contradicted.

Our charter from the State of Illinois authorizes us to teach the true art of healing, and to those qualified grant diplomas, conferring the degree of M. D. the same as any other American or European medical college, and in addition, we act in connection with a medical society which grants certificates of qualification to those particularly well qualified. Thus we believe bringing them fully up to all the present legal requirements in some eight or ten great States. We also further believe that where one has fully complied with all the legal requirements in any State, no law could be held constitutional that would disqualify them for practice. We enclose you our annual announcement, and will be pleased to answer any questions not answered in same. To those who have never studied medicine, the opportunities we offer deserve careful consideration.

N. B. We can give parties desiring to avail themselves of these advantages the benefit of them without the expense of a journey here. The questions on the sheet can be answered in any of the following languages: English, French, German, Spanish, Italian, Dutch, Swedish, Norwegian, Danish, Flemish, Bohemian, Russian, Japanese, Malay or Arabian.

ILLINOIS HEALTH UNIVERSITY, Chicago.

[The idea of a "splendidly equipped physician" without a diploma is good.—ED.]

### IMPORTANT TO CANDIDATES DESIRING TO GRADUATE.

We enclose you a list of questions, please answer and return with affidavit as directed.

If you can not answer all of said questions, kindly answer what you can to the best of your knowledge and ability. This will place us in a position to judge of your qualifications and inform you what books will be needed, and the length of time required to qualify you for graduation.

A great many men and women have, by reading and practice at home, acquired a better medical education than M. D.'s who have spent years in college. Send proof that you have practiced successfully and this will excuse you answering many of the questions. Those that have not made any preparation will require considerable time to fit them for the practice of medicine, but by the proper course of study, and reading the proper books, they can be fully equipped much sooner than many suppose.

Following are a list of said questions:

Define:—Anatomy, physiology, hygiene, materia medica, therapeutics, obstetrics and surgery, and mention ten important facts relating to each branch that a physician should know in order to be a successful practitioner; also give reasons substantially proving that the facts mentioned are of genuine benefit, and absolutely essential in the treatment of the different forms of disease.

To illustrate—A student might be able to name every bone and muscle in the body and make an entire failure in treating simple cases, while one who did not possess one-tenth of said knowledge might succeed. We want you to possess the knowledge *that will enable you to cure your patients.* No amount of superfluous nonsense so extensively taught in medical monopoly colleges and called "medical science" will fill the bill.

Carefully dissect every fact given in your answers, and ask yourself the question, will said knowledge *actually* enable me to cure my patients, if not, what knowledge can I acquire that will, and what is the quickest way for me to acquire it?

Outline your treatment for the following forms of disease, and state what medicines, "if any," you would give in such cases, or on what you would chiefly rely to affect a cure: Asthma, bronchitis, cancer in the first stages, croup, diarrhea, diphtheria, dropsy, dysentery, dyspepsia, epilepsy, erysipelas, inflamed eyes, fits in children, gravel, headache, hip joint disease, hydrocele, hysteria, insanity in the first stages, typhoid fever, jaundice, malaria, neuralgia, paralysis, piles, pleurisy, polypus, nervous prostration, rheumatic fever, rheumatism, sciatica, scrofula, sleeplessness, St. Vitus dance, ovarian tumors, fibrous tumors, or any other forms of disease you have treated, or think you can treat successfully.

Name ten remedies that you consider good medicines to give to the sick, and describe their medical properties. On what do you chiefly rely to affect cures?

What do you understand by the allopathic system of medicine, and do you approve of it? If not, why? What system do you approve of? Answer same questions in regard to the homeopathic, the eclectic, physio medical and hydropathic systems.

Do you consider it in accordance with science, reason or common sense to give to the sick such agents as arsenic, strychnine, morphine, calomel, aconite, digatalis and the numerous poisons so extensively used in medical practice?

State what medical books you now own and have studied, length of time you have spent in the study of medicine at home, under a preceptor, or in a medical college. Name college or colleges attended, if any, and state any other facts going to show what opportunities you have had to become a competent practitioner.

If you have practiced, state number of years, and send, if you can, sworn statements from patients to the effect that you have treated them successfully, especially send affidavits of cures effected by you after "so-called" highly educated medical monopoly college graduates failed.

State name of notary public before whom you are willing to go and answer another list of questions on the treatment of different forms of disease without having access to said questions. State if you consider yourself qualified to conduct a safe practice. If you will carefully, of your own knowledge "alone," answer said questions to the best of your ability, and we should consider you deficient in some respects, we will carefully point out said deficiency, and how to acquire the knowledge you lack in the shortest time.

Send letters of recommendation from three or more prominent and responsible citizens as to your moral standing, and if you have practiced, as to your professional ability. Have answers type-written if convenient, if not write plainly on foolscap, and make affidavit as follows:

I (name in full) do solemnly swear that the foregoing are my answers to the questions asked me by the faculty of the Illinois Health University, and are given of my own knowledge without copying from books or without any assistance but my own mental faculties, and that said knowledge has been acquired by a careful study of medical works and by my own experience, and that the other statements therein contained are true to the best of my knowledge and belief.

Have statements and answers sworn to before notary public and mailed to us.

J. ARMSTRONG, M. D., President.

These documents are accompanied by the cut of a wild-eyed, slick, bald-headed young man of about 30.

Here is the catch penny:

For full particulars how to become lawful physicians if qualified, or how ladies or gentlemen employed day time can acquire a medical education without patronizing medical monopoly colleges. Course by mail. Address,

ILLINOIS HEALTH UNIVERSITY.

M. L. Reed, M. D., Secretary.

S. Armstrong, M. D., President.

What do our readers think of this scheme? And who says we do not need medical legislation? These sharpers ought to be in the penitentiary picking oakum.

This is a pretty turning of the tables. It is a just cause of complaint that a graduated and capable physician, who has expended time and money on a medical education, has to compete with the verriest ignoramus; but these fellows put the complaint the other way—"the fact leaks out, started by some competitor who has a diploma," that "the doctor has no diploma, and he [the fellow without a diploma] finds his business [quackery] seriously crippled." Well, I should say so. And yet this clap-trap will catch its hundreds of the gullible. Alas for the honor and dignity of the once "noble profession." And it would seem that there is no law to reach these fellows who grant diplomas *in absentia*. What a commentary on our statutes, and those who make them.

The above is published as a curiosity as well as a warning.

TEXAS "HEALTH" COLLEGE DIPLOMAS:—In response to our request, a number of our subscribers have sent us the name and address of parties practicing medicine by authority (?) only of a diploma from the "Myth of the Mound," the alleged Texas Health College, claimed to be located at Mound, Coryell county. We have presented the names to Attorney-General Crane, who will, in pursuance with his promise to us, call the attention of the district attorney to them in the respective districts where these parties reside, and instruct them to present the offenders to the grand jury. So we may look to hear something drop.

